# Identity by descent mapping of HCV spontaneous clearance in populations of diverse ancestry

**DOI:** 10.21203/rs.3.rs-2433454/v1

**Published:** 2023-01-09

**Authors:** Zixuan Yu, Salma Abdel-Azim, Priya Duggal, Candelaria Vergara

**Affiliations:** Johns Hopkins University, Bloomberg School of Public Health; Johns Hopkins University, Bloomberg School of Public Health; Johns Hopkins University, Bloomberg School of Public Health; Johns Hopkins University, Bloomberg School of Public Health

**Keywords:** IBD mapping, HCV clearance, rare variants, GWAS, unrelated individuals

## Abstract

**Background:**

Acute infection with hepatitis C virus (HCV) affects millions of individuals worldwide. Host genetics plays a role in spontaneous clearance of the acute infection which occurs in approximately 30% of the individuals. Common variants in *GPR158*, genes in the interferon lambda (*IFNL*) cluster, and the MHC region have been associated with HCV clearance in populations of diverse ancestry. Fine mapping of those regions has identified some key variants and amino acids as potential causal variants but the role of rare variants in those regions and in the genome, in general, has not been explored. We aimed to detect haplotypes containing rare variants related to HCV clearance using identity-by-descent (IBD) haplotype sharing between unrelated cases/case pairs and case/controls pairs in 3,608 individuals with European and African ancestry.

**Results:**

We detected 1,711,832 and 5,678,043 and individual pairs of IBD segments in the European and African ancestry individuals, respectively. As expected, individuals of African descent had more, and shorter segments compared to Europeans. We did not detect any significant IBD signals in the known associated gene regions.

**Conclusions:**

IBD is based on sharing of haplotypes and is most powerful in populations with a shared founder or recent common ancestor. For the complex trait of HCV clearance, we used two outbred, global populations that limited our power to detect IBD associations. Overall, in this population-based sample we failed to detect rare variations associated with HCV clearance in individuals of European and African ancestry.

## Background

Hepatitis C virus (HCV) infection affects around 200 million individuals worldwide [1, 2]. Approximately 30–50% of HCV-infected individuals spontaneously clear the virus[3–5] while others will experience persistent HCV infection developing chronic hepatitis C, liver cirrhosis, and HCV-related hepatocellular carcinoma [6]. The proportion of individuals who spontaneously clear HCV varies by ancestry[7] and is influenced by age and comorbidities, especially other viral infections including HIV or HBV co-infections.

Host genetics is a key determinant of both spontaneous clearance of HCV infection as well as anti-viral treatment response [3]. Previous genome-wide association studies (GWAS) have identified common variants with large effect in the region of *IFNL4* and *IFNL3* genes, the MHC region and G-protein-coupled receptor 158 gene (*GPR158*) in populations of diverse ancestry [8, 9]. Collectively, these variants with high minor allele frequency explain 5–7% of the variance of HCV clearance across ancestries [9, 10]. Fine mapping of the MHC signal suggested amino acids in the HLA-DQβ1 molecule as potential causal variants in the region for both populations [11]. In contrast, limited results were found in the effort to fine map the *IFNL* locus by sequencing, attributable to a genomic structure with high repetitive segments in that region [12].

The role of single rare variants potentially contributing to HCV clearance variability is unknown and often difficult to explore without sequencing a substantially large number of samples. As an alternative, identity-by-descent method (IBD) leverages genome wide array data to detect haplotype sharing and identifying signals with rare disease-causing variants. This method determines whether two purportedly unrelated pairs of individuals in a dataset share segments at a certain genomic position inherited from a common ancestor and, by using “pairwise” statistics, the rate of IBD in case/case pairs is compared to that in case/control pairs[13–17] to detect segments with excess of sharing among cases. This method has successfully detected rare variants for non-infectious diseases related complex traits such as diabetes, acne, multiple sclerosis, and schizophrenia[16, 18–20] as well as ultra-rare loss-of-function variation associated with blood-related traits in individuals from the UK Biobank dataset [21]. We aim to detect the effect of rare variation using IBD mapping in a large dataset of unrelated individuals of European and African descent with HCV clearance and persistence.

## Results

### Study Sample

We performed IBD mapping using GWAS data for HCV clearance and persistence from a sample consisting of 3,608 individuals from two genetically determined ancestry groups participants of the Extended HCV Genetic consortium, as previously described [8, 9, 22]. This includes 1,869 individuals of African ancestry (340 with HCV clearance and 1,529 with HCV persistence) and 1,739 persons of European ancestry (702 with HCV clearance and 1,037 with HCV persistence). Distribution of the analyzed individuals by genetically determined ancestry group, sex, and HIV infection status is presented in Table 1. The two groups are ancestrally distinct, and no genetic structure was present in either group (Supplementary Figure S1).

### Genotyping and IBD mapping

**Figure 1:**
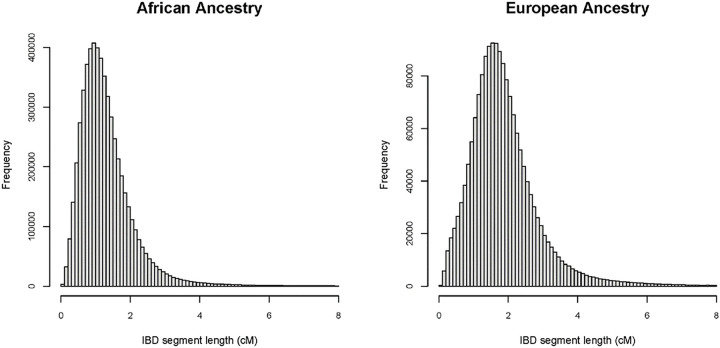
Distribution of the lengths of detected IBD segments in individuals with HCV clearance and persistence from populations of African ancestry (Left Panel) and European ancestry (Right Panel).

After standard GWAS quality control [8], we used 661,397 autosomal single nucleotide polymorphisms (SNPs) to detect IBD segments in cases and controls. After the union of the overlapping IBD segments, we detected 1,711,832 and 5,678,043 unique segments < 8 cM in the European and African ancestry group, respectively. For the analysis, we used 585,130 and 3,796,732 case/case IBD pairs in the European and African ancestry populations, respectively and 829,906 and 1,693,552 case/control pairs in those populations. Distributions approximated a Pareto distribution with the mean lengths of segments of 1.9 cM for the European ancestry and 1.3 cM for the African ancestry group. Distribution in the African population showed a higher number of segments < 1cM in comparison with the European population (Fig. 1).

**Figure 2:**
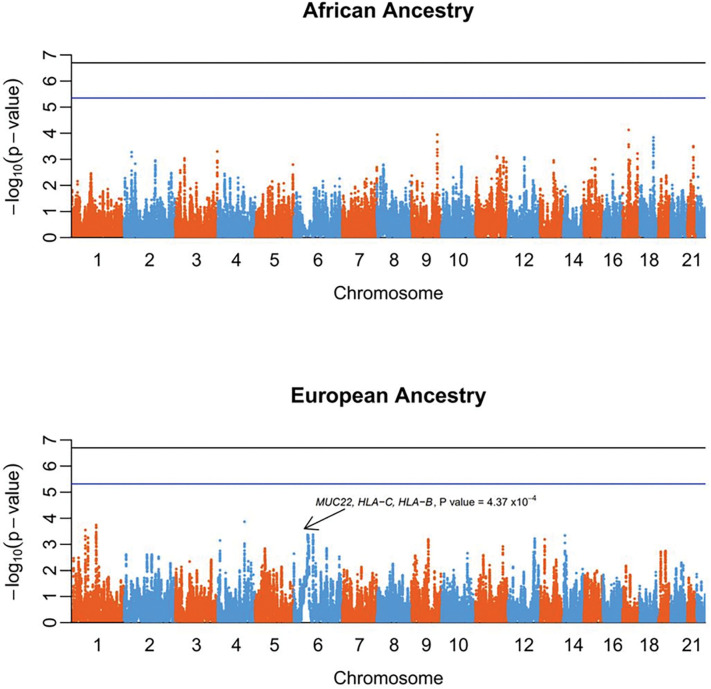
Manhattan plot of the P values obtained using IBD permutation analysis in individuals of African (Upper Panel) and European ancestry (Lower Panel). The blue line indicates the genome-wide threshold for each population and the black line is the minimal permutation value obtainable with 5 million permutations (2×10^−7^). Each dot corresponds to the P value of a genetic marker. Chromosome coordinates are based on GChR37/hg19 built.

Segments with an IBD probability P value < 1×10^− 10^ were analyzed using a permutation analysis comparing the rate of IBD shared between case/case to that between case/control and to identify IBD alleles in SNPs contained in those segments in each ancestry population. This analysis failed to confirm known associated regions or detect new regions with significative association at GWAS level in both populations. However, we observed one suggestively associated locus in the European ancestry population located in the MHC region (chr6p21.33), 7.8 kb downstream from the mucin 22 (*MUC22*) gene where the top marker was rs2517549 (6:31008598, C > A, P value = 4.37×10^− 4^). This IBD region extends ~ 257 kb downstream from the *HLA-C* and *HLA-B* genes (Fig. 2 and Supplementary Figure S2) and, is concordant with the association previously identified using traditional SNP based GWAS [8].

## Discussion

In this study, we performed IBD mapping of HCV spontaneous clearance in a European and African ancestry populations and did not identify any significant associations. We identified a suggestive association with sharing of haplotypes the MHC region in individuals of European ancestry in concordance with previous results obtained using a GWAS approach.

IBD mapping is advantageous at detecting variants where there are multiple rare causal variants clustered within a gene, and in this scenario, it is well powered at identifying significant genetic regions [16]. However, the ability to detect IBD depends on the number of generations to the common ancestor which is reflected by the length of the IBD segments. More recent common ancestry tends to result in longer and more detectable IBD segments [23]. As expected, in this study we identified longer segments in the European ancestry population as compared to the African ancestry population and even though the results were not significant, the analysis in the European ancestry population was able to detect suggestive signals in the MHC complex in concordance with previous SNP based analysis.

Additionally, simulation data indicates that the relative performance of IBD mapping and SNP association testing depends on population demographic history as well as the strength of selection against causal variants. For outbred populations, very large sample sizes may be required for genome-wide significance unless the causal variants have strong effects. We consider that the inability to detect strongly associated regions in this study is likely due to the large diversity of the haplotypes present in these outbred populations especially in the admixed population of African descent [24]. Moreover, hepatitis C viral infection does not exert a large selective pressure in the host genome to create large conserved and detectable haplotypes shared by cases [25, 26]. Hepatitis C infection is often acquired after childhood thus not affecting the fitness of host alleles. This is in comparison to other infections such as *Plasmodium falciparum* malaria which exerts strong selective pressure on the human genome[27] and results in conserved regions across ancestral populations with a higher homogeneity of haplotypes susceptible to be detected by IBD in unrelated individuals [28]. This difference in pathogen pressure likely limits the utility of IBD for infectious diseases that occur globally, and thus in outbred populations, but without strong selective pressure.

One of the strengths of this study is the IBD analysis in populations of African ancestry with an astringent methodology, which highlights (with actual data) the need for larger samples sizes to detect any associations in this genetically diverse population in comparison with populations of European ancestry.

## Conclusions

IBD mapping is an alternative to sequencing for prioritizing both individual samples and genomic regions harboring rare variants for follow-up analysis. IBD mapping for HCV clearance suggested associations with previously identified regions in individuals of European ancestry and highlighted the need for larger sample sizes especially in populations with shorter and more diverse IBD segments with low pathogen selective pressure.

## Methods

### Study population

In this study, we analyzed 3,608 individuals from 2 genetically determined ancestry groups participants in the Extended HCV Genetics Consortium [8, 9, 22]. This is a multi-site international consortium including multiple studies from Europe and United States in which HCV infection outcomes were ascertained. Information about HIV infection status was also obtained in the included individuals since it is a determinant of HCV clearance. Each individual study obtained consent for genetic testing from their governing Institutional Review Board (IRB) and the Johns Hopkins School of Medicine Institutional Review Board approved the overall analysis [8].

### Genotyping:

Genotyping and quality control has been detailed in [8]. Briefly, samples were genotyped using the Illumina Omni1-Quad BeadChip array (Illumina) and processed using standard genome-wide association study protocols for quality control [8]. In this study, only autosomal SNPs were used for detecting IBD segments and markers in mitochondrial DNA and sex chromosomes were disregarded. Genetic ancestry and population structure for the Extended HCV Genetic consortium was determined by principal component analysis (PCA) using the smartpca program in EIGENSOFT [29] indicating no significant population structure between cases and controls in each ancestry group (Supplementary Figure S1).

### Transformation of datasets and IBD mapping

Datasets for each ancestry were transformed from PLINK to Beagle’s .bgl format using PLINK [13]. We used Beagle version 3.3 to phase the data and FastIBD for detecting IBD segments[16] in the complete set of cases and controls in each ancestry separately. FastIBD finds tracts of identity by descent between pairs of individuals. It estimates frequencies of shared haplotypes based on genome-wide SNP data considering that a rare, shared haplotype is likely to be identical by descent. This method allows for uncertain haplotype phases by sampling multiple realizations of haplotype phase given the data, then allowing for some switching between alternative phasing with a penalty to prevent excessive switching. The extent of haplotype sharing is measured by a score that is the frequency of the shared haplotype modified by the switching penalties assessed at each switch between alternate phasings [16]. The genetic and physical distances were based on build GRCh37/hg19 of the human genome [30]. The centimorgan distances for the .map files for each chromosome were interpolated using the Beagle utility program base2genetic.jar [16]. We did 10 FastIBD runs for each chromosome starting with a random seed generator. The output of the IBD calculations is a series of potential IBD segments shared between a pair of individuals containing the information of the first and last SNP of the segment, length of the segment in centimorgans and the probability of the two individuals both carrying the segment if it was not IBD. We filtered the segments using a threshold of 1×10^− 10^ before calculating the genome wide average and performing the permutation analysis to compare the IBD rates between pairs. Publicly available scripts[15] were used to combine the IBD segments that reached the threshold by taking the union of the IBD segments detected in each run. Scripts were also used to perform the IBD test, calculated as the difference in IBD proportions between case/case and case/control pairs and compared this difference to that obtained from 5 million permutations of case-control status. Because of the limited number of permutations, the smallest P value detectable is 2×10^− 7^. We calculated the difference in IBD proportions and the corresponding permutation P-value at every 10th SNP along the autosomes. In addition, we calculated permutation P-values genome-wide for 1000 permutations of case-control status, which allows us to determine the correct multiple-testing adjustment. The threshold for genome-wide significance was estimated as the 0.05 percentile of the distribution for the permutation P-values corresponding to P value = 4.83 × 10^− 6^ and 4.47 × 10^− 6^ for the European and African and ancestry populations, respectively. These values are very similar to previously established genome-wide significance threshold for IBD mapping using a population of European ancestry [15]. When calculating the P values in the permutation analysis, it was corrected for the average genome-wide sharing as recommended by the authors [15]. We used customized R scripts to graph the distribution of the length of the detected IBD segments, and to examine the distribution of IBD P values among the 22 chromosomes. Locus Zoom [31, 32] was used for the visualization of regions with suggestive signals and to anatomize regions of interest.

## Figures and Tables

**Table 1:** Demographic characteristics of the analyzed studies by genetically determined ancestry groups.

Genetically Determined Ancestry Group	N	HCV infection Persistence:Clearance	(+) HIV infection	Female Sex
African ancestry	1869	1529:340	38%	33%
European ancestry	1739	1037:702	16%	31%
Total	3608	2566:1,042	28%	34%

## Data Availability

Genotype data is available upon request at dbGaP with accession number phs000454.v1.p1. Python programs implementing the IBD test that we used for the HCV clearance data can be downloaded from http://faculty.washington.edu/sguy/ibdmapping.html.

## Abbreviations

IBD: identical by descent
GWAS: Genome wide association study
HCV: Hepatitis C virus
PCA: principal component analysis
MUC22: mucin 22
